# Fasting as an intervention to alter the impact of simulated night-shift work on glucose metabolism in healthy adults: a cluster randomised controlled trial

**DOI:** 10.1007/s00125-024-06279-1

**Published:** 2024-10-18

**Authors:** Stephanie Centofanti, Leonie K. Heilbronn, Gary Wittert, Jillian Dorrian, Alison M. Coates, David Kennaway, Charlotte Gupta, Jacqueline M. Stepien, Peter Catcheside, Crystal Yates, Linda Grosser, Raymond W. Matthews, Siobhan Banks

**Affiliations:** 1 Behaviour-Brain-Body Research Centre, UniSA Justice and Society, University of South Australia, Adelaide, SA Australia; 2 Lifelong Health Theme, South Australian Health and Medical Research Institute (SAHMRI), Adelaide, SA Australia; 3 Faculty of Health and Medical Sciences, University of Adelaide, Adelaide, SA Australia; 4 The Freemasons Centre for Male Health and Wellbeing, University of Adelaide, Adelaide, SA Australia; 5 Alliance for Research in Exercise, Nutrition and Activity, University of South Australia, Adelaide, SA Australia; 6 Appleton Institute, Central Queensland University, Rockhampton, QLD Australia; 7 Flinders Health and Medical Research Institute: Sleep Health (formerly Adelaide Institute for Sleep Health), Flinders University, Adelaide, SA Australia

**Keywords:** Glucose metabolism, Meal size, Meal timing, Obesity, Shift work, Time-restricted eating, Type 2 diabetes

## Abstract

**Aims/hypothesis:**

Night-shift work causes circadian misalignment and impairs glucose metabolism. We hypothesise that food intake during night shifts may contribute to this phenomenon.

**Methods:**

This open-label, multi-arm, single-site, parallel-group controlled trial involved a 6 day stay at the University of South Australia’s sleep laboratory (Adelaide, SA, Australia). Healthy, non-shift-working adults without obesity (*N*=55; age 24.5 ± 4.8 years; BMI 24.8 ± 2.8 kg/m^2^) were assigned to the next available run date and cluster randomised (1:1:1) to fasting-at-night (*N*=20), snack-at-night (*N*=17), or meal-at-night (*N*=18) conditions. One participant withdrew from each group, prior to starting the study. Due to study design, neither participants nor people collecting their measurements could be blinded. Statistical and laboratory staff were concealed to study allocation. Participants were fed at calculated energy balance, with the macronutrient composition of meals being similar across conditions. The primary outcomes were a linear mixed-effects model of glucose, insulin and NEFA AUC in response to a 75 g OGTT that was conducted prior to and after 4 consecutive nights of shift work plus 1 night of recovery sleep. Insulin sensitivity, insulinogenic and disposition indexes were also calculated.

**Results:**

Night-shift work impaired insulin sensitivity, as measured by insulin AUC (*p*=0.035) and the insulin sensitivity index (*p*=0.016) across all conditions. Insulin secretion, as measured by the insulinogenic index, was increased in the fasting-at-night condition only (*p*=0.030), resulting in a day×condition interaction in glucose AUC (*p*<0.001) such that glucose tolerance was impaired in the meal-at night (+2.00 [95% CI 1.45, 2.56], *p*<0.001) and snack at-night (+0.96 [0.36, 1.56], *p*=0.022) conditions vs the fasting-at-night (+0.34 [–0.21, 0.89]) condition. A day×condition interaction was also observed in NEFA AUC (*p*<0.001), being higher in the meal-at-night (+0.07 [0.03, 0.10]. *p*=0.001) and snack-at-night (0.01 [–0.03, 0.05], *p*=0.045) conditions vs the fasting-at-night condition (–0.02 [–0.06, 0.01]). No adverse events occurred.

**Conclusions/interpretation:**

The timing of food intake has a critical effect on glucose metabolism during simulated night-shift work, which was readily amendable to a meal re-timing intervention.

**Trial Registration:**

Australian New Zealand Clinical Trials Registry (ANZCTR) ACTRN12616001556437

**Funding:**

This work was funded by the National Health and Medical Research Council (NHMRC), APP1099077.

**Graphical Abstract:**

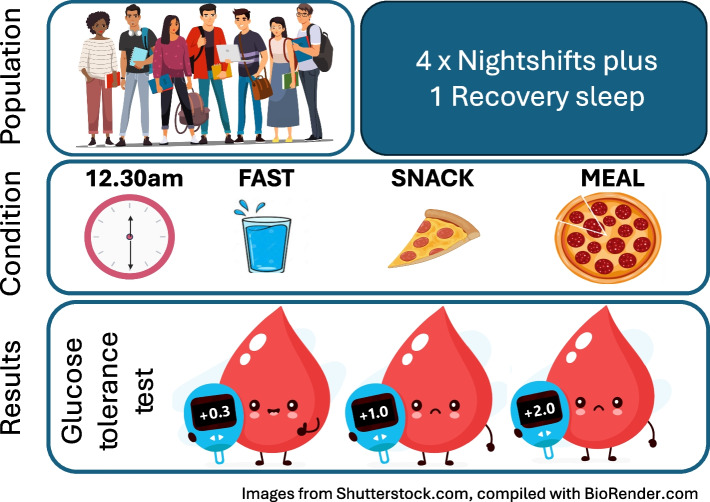

**Supplementary Information:**

The online version of this article (10.1007/s00125-024-06279-1) contains peer-reviewed but unedited supplementary material.


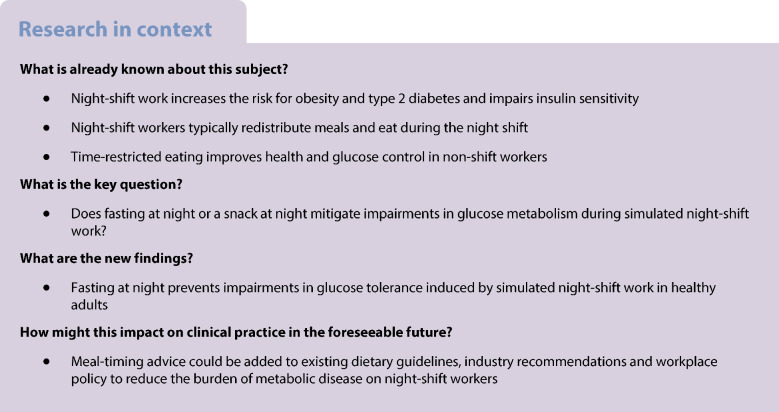


## Introduction

Around 20% of the working population is required to work outside the regular working day (09:00–17:00 hours) and this number is increasing as economic demands push work hours into the night for many industries [1, 2]. Irregular schedules mean that shift workers often sleep during the day and are awake at night, resulting in a misalignment between the normal day/light-entrained internal physiological processes and the external environment. Night-shift workers gain more weight after starting their jobs [3], have higher rates of obesity and type 2 diabetes (even after adjusting for lifestyle and socioeconomic circumstances), and those with type 2 diabetes have poorer glucose control [4–7]. However, night-shift workers also alter their eating patterns, habitually eating during the night shift [8, 9]. This transferral in eating behaviours could contribute to increased disease risk.

In non-shift workers, meals consumed after 20:00 hours are associated with increased weight, even after controlling for sleep timing and duration [10]. Eating later in the day reduces the effectiveness of weight-loss programmes, independently of energy intake, dietary composition or sleep [11]. Thus, time-restricted eating (TRE), whereby food is consumed ad libitum throughout a restricted daytime period, has emerged as a novel therapeutic intervention to improve glucose and lipid metabolism in non-shift-working adults [12]. In rodents, night-shift work is simulated by periodically rotating the light–dark cycle. The metabolic consequences of simulated shiftwork in rodents are lessened by TRE [13], even when the animals are provided with high-fat diets [14]. While these results are promising, successful translation of these basic biological data into improved outcomes in shift-working humans is lacking.

In a controlled laboratory setting, we conducted a small study (*N*=4–7/condition) that showed that not eating at night prevented impaired glucose tolerance after 4 days of simulated night-shift work [15]. This suggested, for the first time, that glucose disturbance in shift workers was strongly mediated by the temporal distribution of meals across the day and night. Another study subsequently extended this finding to a simulated rotating shiftwork schedule [16]. Thus, restricting eating to the day may prevent the misalignment between central and peripheral circadian rhythms that occur in simulated night work [17].

To what extent the size of a night-time meal plays a role in this remains to be determined. It is important to resolve this, as redistributing the usual 24 h energy intake to only the daytime has notable practical implications. While one study identified that fasting during a night shift is feasible [18], many night-shift workers report snacking during the night shift [19] and may find it difficult to completely abstain from eating. In a pilot trial, we showed that consuming a small snack (840 kJ) at midnight during one simulated night shift did not impair glucose tolerance the following morning [20]. Thus, allowing a night-time snack would be both feasible and effective at mitigating glucose-metabolism impairment during night-shift schedules.

The aim of this three-arm, parallel-group, cluster randomised controlled trial was to examine the effects of eating a meal at night vs a snack at night vs fasting at night on glucose metabolism during simulated night-shift work in healthy non-shift-working adults. We hypothesised that fasting at night and eating a snack at night would enable maintenance of glucose tolerance following exposure to simulated night-shift work as compared with eating a meal at night.

## Methods

### Study details

**Fig. 1:**
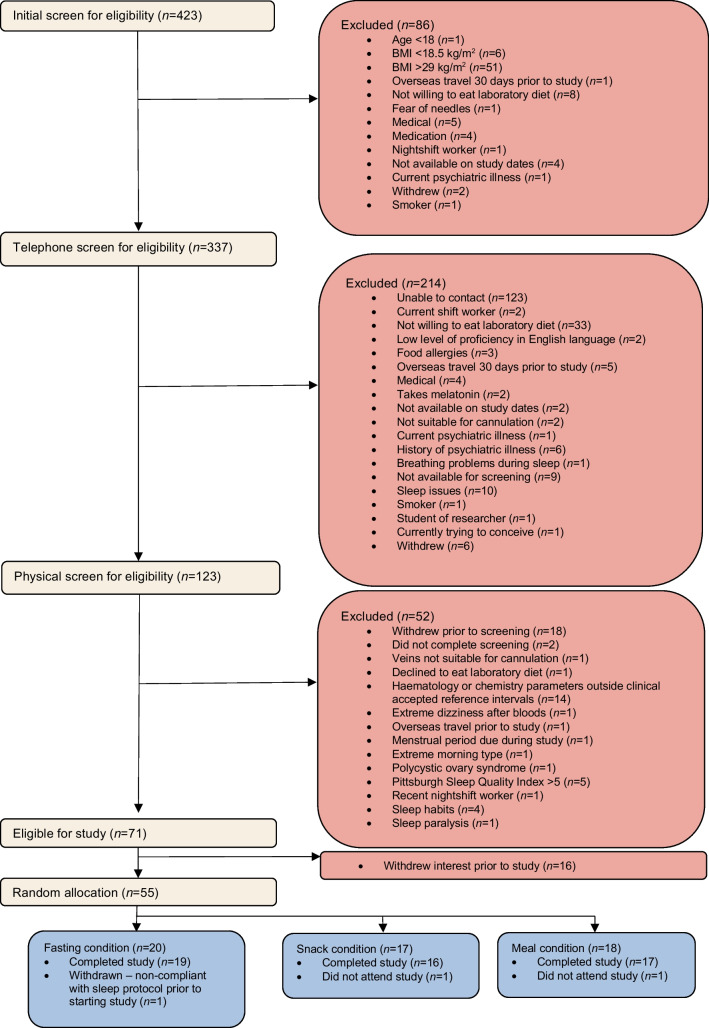
Participant flow diagram showing the screening and recruitment process, and the final eligible study participants who were randomised, reasons for withdrawal, and numbers included in the final analysis set

An experimental, three-condition, between-group study design was used (electronic supplementary material [ESM] Fig. 1). The three conditions used were meal-at-night, snack-at-night and fasting-at-night conditions. The primary outcomes were glucose, insulin and NEFA AUC in response to an OGTT following 4 nights of simulated shift work plus 1 night of recovery sleep as compared with baseline. Exploratory outcomes were: (1) fasting and postprandial glucose and insulin responses to a high carbohydrate breakfast meal after the first and fourth night of shiftwork; and (2) sleep health at baseline and following the third night of shiftwork and during the recovery sleep, assessed by polysomnography (PSG). The study was conducted between 28 June 2016 and 4 March 2019 and approved by the University of South Australia Human Research Ethics Committee (no. 0000033621). It was conducted according to the Consolidated Standards of Reporting Trials (CONSORT) guidelines and registered with Australian New Zealand Clinical Trial Registry (ANZCTR; Australian clinical trials registration number (ACTRN) registration no. ACTRN12616001556437).

### Participant recruitment and screening

#### Study participants

Healthy, non-shift-working participants, aged 18–50 years (32 men, 23 women, self reported) were recruited from the general population via flyers and online advertisements. Race was self-reported with white (56%), Asian (21%), Latino (19%), or mixed race (2%). Latino were over-represented as compared to the general population. Exclusion criteria were the presence of self-reported medical or psychiatric disorders, or sleep-disordered breathing, as assessed by two commonly used questionnaires [21, 22] and confirmed by PSG. Participants were also required to have habitual sleep duration >7 h and <9 h, score <5 on the Pittsburgh Sleep Quality Index [23] and score between 22 and 43 on the Composite Morningness questionnaire [24]. Participants were excluded if they met any of the following criteria: (1) any haematology or chemistry parameters outside of clinically accepted reference intervals, as assessed by a screening blood test ; (2) BMI was outside of the normal to overweight range (18.5–29.9 kg/m^2^); (3) regular medication use other than oral contraception; (4) drug and/or alcohol abuse (>20 g of alcohol per day); (5) >2 h of structured high-impact activity/exercise per week; (6) food allergies; or (7) pregnancy. Trans-meridian travel in the 60 days prior to the study, a history of shiftwork to ensure stable sleep/wake patterns and circadian misalignment were also exclusionary, as is standard in a sleep-laboratory study [15]. Female participants were scheduled to participate in the luteal phase of their menstrual cycle to control for changes in sleep, hormonal factors and basal metabolism.

#### Pre-study conditions

Respondents to advertisements underwent initial telephone screening for eligibility and, after giving informed written consent, they attended two physical screening sessions for more detailed assessment of eligibility. If eligible, participants completed a 7 day sleep diary and wore an activity monitor (Phillips Respironics Actiwatch, Murrysville, PA, USA) to assess sleep patterns at home in the 7 days prior to the laboratory study. Participants were asked to keep a strict sleep schedule (22:00–23:00 hours to 06:00–07:00 hours) with no naps, with adherence subsequently confirmed via actigraphy. They were required to refrain from caffeine, alcohol and over-the-counter medications. To confirm the absence of illicit substances (amphetamines, opioids, benzodiazepines, cannabinoids and cocaine), urine toxicology was performed immediately prior to the laboratory study commencing.

### Study design

#### Laboratory study conditions

The study coordinator allocated two to four participants to each study run based on their availability and order of appearance. All participants within each run cluster were studied simultaneously under identical conditions, with 55 participants assigned to one of 15 study runs. Prior to the start of the study, the study run was randomly assigned to a condition (1:1:1) using a random number generator by the principal investigator, who had no contact with study participants. Upon arrival at the laboratory, one participant was found not to have adhered to the sleep schedule during the lead-in period and was excluded. Two participants were lost to contact during the lead-in period. Thus, 52 participants took part in and completed the trial (Fig. 1).

Participants lived in the controlled laboratory environment at the University of South Australia’s Sleep and Chronobiology Laboratory (Adelaide, SA, Australia) for a 151 h period (7 days). All participants had an 8 h time-in-bed (TIB) sleep opportunity on the first night (ESM Fig. 1) from 22:00–06:00 hours ahead of the baseline day. Participants then transitioned to simulated night work and remained on that schedule for 4 nights (night shift 1–4 [NS1–4]), with a 7 h sleep opportunity from 10:00–17:00 hours on each day. This experimental period was followed by a final night with an 8 h sleep opportunity (TIB: 22:00–06:00 hours) to simulate the return to a daytime schedule (RTDS). Four nights were chosen to examine the cumulative impact of the eating schedules on glucose metabolism because most shiftwork schedules limit the number of consecutive nights worked to 4 or fewer [2].

The ambient temperature was 22±1°C, while light levels were 100 lux during wakefulness to simulate an office work environment and <1 lux during sleep times. Access to clocks or social time cues (i.e. mobile phones, laptops, live television) was not permitted. During discretionary time, participants were able to watch movies, read or converse with staff. They were not able to engage in any exercise or consume stimulants (e.g. caffeine). Meals were strictly timed and controlled, with a similar amount of total energy and macronutrient intake each day across the conditions. On the morning after the baseline sleep and after the RTDS sleep, a 75 g OGTT was conducted. On the morning after NS2 and after NS4, a standardised high carbohydrate breakfast was consumed and glucose and insulin responses to the breakfast were measured. The OGTT is a clinical tool for diagnosis of type 2 diabetes, whereas a standardised meal provides a more physiologically relevant measure of meal-related metabolic responses [25]. Due to the design of the study, neither participants nor study staff were blinded to study conditions during data collection, but laboratory staff and statistical analysis were performed blinded to study condition.

#### Meal conditions

Participants consumed meals at specific times depending on the condition. Water and caffeine-free tea were allowed ad libitum. Each meal and snack were similar across conditions and were comprised of approximately 40% carbohydrate, 33% fat, 17% protein and 23 g fibre. Participants were encouraged to consume all food they were given within 15 min (breakfast), 30 min (snacks and lunch), or 45 min (dinner). Daily estimated energy requirements (EER) were individually calculated by validated reference equations, rounded to the nearest 500 kJ, and reduced by 15% to allow for the extremely sedentary activity levels in the laboratory. Meal plans were analysed using Foodworks version 8 (Xyris, Spring Hill, QLD, Australia). On entry to the laboratory, participants consumed lunch at 12:00 hours (30% of 24 h energy intake) and dinner at 19:00 hours (40% of 24 h energy intake). On RTDS, all participants consumed a snack at 10:00 hours (10% of 24 h energy intake) and lunch at 12:00 hours (40% of 24 h energy intake) before leaving the laboratory. During the simulated shiftwork period, timing of food intake per 24 h depended on the study condition. In the meal-at-night condition, participants ate at 19:00 hours (dinner; 40% EER), 00:30 hours (lunch; 30% EER) and approximately 06:30 hours (breakfast; 30% EER; timing similar to OGTT at baseline). In the snack-at-night condition, participants ate at 19:00 hours (dinner; 40% EER), 00:30 hours (snack; 10% EER), approximately 06:30 hours (breakfast; 30% EER) and 17:00 hours (snack; 20% EER). In the fasting-at-night condition, participants consumed food at 19:00 hours (dinner; 40% EER), approximately 06:30 hours (breakfast; 30% EER), 09:30 hours (snack; 10% EER) and 17:00 hours (snack; 20% EER). At baseline and RTDS, all participants completed an OGTT, with the glucose drink accounting for 10% EER.

#### Biological sampling

After an overnight fast, at baseline and on the mornings after the RTDS sleep, an in-dwelling catheter was inserted in the median cubital vein at 06:15 hours, and blood was sampled before and after a 75 g glucose drink (at −15 min, 0 min, 30 min, 60 min, 90 min, 120 min and 150 min) and assayed for glucose, insulin and NEFA levels. On the morning following NS1 and NS4, an in-dwelling catheter was inserted in the median cubital vein at 06:15 hours and blood was sampled before and after (at −15 min, 0 min, 30 min, 60 min, 90 min, 120 min and 150 min) a high carbohydrate breakfast, comprising approximately 76.0% carbohydrate, 13.8% protein, 7.5% fat and 2.6% fibre [26], and assayed for glucose and insulin levels. Sample lines were occasionally lost and not able to be replaced, or the sample volume obtained was too small; exact *n* values available for each assessment is included in the table footnotes.

Plasma glucose was analysed using a commercial assay kit, InfinityTM Glucose Hexokinase Liquid Stable Reagent (Thermo Scientific, Middletown, VA, USA), with the assay being performed on the Konelab 20XT (Thermo Scientific, Middletown, VA, USA). Intra- and inter-assay CVs for the assay were 4.5% and under 2.0%, respectively. Insulin was measured by radioimmunoassay (HI−14K; Millipore, Billerica, MA, USA) at the Adelaide Research Assay Facility (University of Adelaide, Adelaide, SA, Australia). Samples were assayed directly as per the manufacturers’ protocol. The intra-assay CV was 4.9%, while the inter-assay CV was 11.6% at the low-quality control level and 37.7% at the high-quality control level.

Sleep quality and quantity were recorded using PSG (Compumedics Grael Recorders, Australia) during the baseline-night sleep opportunity (TIB: 8 h), the daytime sleep following NS3 (TIB: 7 h), and the RTDS sleep opportunity (TIB: 8 h). PSG electrode placements were at the F3, F4, C3, C4, O1 and O2 sites referenced to a contralateral mastoid (M1, M2). PSG data were analysed using Rechtschaffen and Kales sleep stage scoring criteria [27]. On the baseline night, respiratory measures (scored according to American Academy of Sleep Medicine criteria) [28] were also used to confirm absence of sleep-disordered breathing, which no participants exhibited. Variables analysed were total sleep time (TST), wake after sleep onset (WASO), sleep efficiency, sleep-onset latency (SOL), and the total time (in min) of rapid eye movement (REM), stage 1, stage 2, stage 3 and stage 4 sleep.

### Statistical analyses

Analyses were completed using IBM SPSS Statistics, version 26.0. (IBM, Armonk, NY, USA). In all analyses, results were considered statistically significant if the *p* value was <0.05. AUC was calculated using the trapezoidal estimation method for 150 min [29]. The oral insulin sensitivity index (ISI), insulinogenic beta cell index (IGI) and disposition index were calculated according to previously validated methods [30, 31]. ISI was calculated using the following equation:

$$\text{10,000}\div \surd (\text{fasting glucose }0\times \text{fasting insulin }0)\times (\text{mean glucose during OGTT}\times \text{mean insulin during OGTT})$$

where 0 refers to timepoint 0 (prior to drinking glucose).

The IGI was calculated using the following equation:

$$(\text{insulin }30-\text{insulin }0)/(\text{glucose }30-\text{glucose }0)$$

where 0 refers to timepoint 0 and 30 refers to timepoint at 30 min.

The disposition index was calculated using the following equation:

$$\text{ISI}\times \text{IGI}$$

Based on an *α* value of 0.05 and the effect size from a previously published pilot study [20], we estimated that 52 participants would be required to detect a significant day×condition interaction, and condition and day effects with 80% power for the three primary outcomes, which were prespecified as change in postprandial glucose, insulin and NEFA AUC in response to the OGTT (ANZCTR registration no. 12616001556437). All other outcomes, including fasting glucose, fasting insulin, postprandial glucose and insulin AUC in response to a breakfast meal after the first and fourth night of shiftwork, and PSG-assessed sleep health at baseline, following NS3 and RTDS, are considered exploratory and thus not adjusted for multiplicity. A linear mixed-model analysis was conducted for each outcome variable with a fixed effect of condition, day and their interaction, and a random effect of participant on the intercept. Results from the models presented include *F* values, *df* (with Satterthwaite-adjusted, rounded denominator) and *p* values for the fixed effects, and the estimated change from baseline with 95% CIs by condition. When significant condition×day interactions were found, post hoc comparisons were conducted for each condition and between conditions. For sleep variables, when a significant effect of day was observed, pairwise comparisons were also conducted for day effects (at baseline, NS3 and RTDS).

## Results

Participant demographics at baseline are shown in Table 1. As shown in the CONSORT diagram (Fig. 1), 52 participants completed the study and were included in the analyses; *n*=19 in the fasting-at-night condition, *n*=16 in the snack-at-night condition and *n*=17 in the meal-at-night condition. No serious adverse events occurred.

**Table 1:** Demographic information by condition at baseline, prior to starting shiftwork

Variable	Fasting-at-night	Snack-at-night	Meal-at-night
Participants (*n*)	19	16	17
Sex (male/female)	11/8	8/8	10/7
Age (years)	24.7 ± 5.4	25.4 ± 5.6	23.5 ± 3.5
BMI (kg/m^2^)	23.9 ± 2.3	23.6 ± 1.9	24.6 ± 3.2
Fasting glucose (mmol/l)^a^	5.2 ± 0.5^a^	5.4 ± 0.6^b^	5.3 ± 0.5
2 h glucose (mmol/l)	6.4 ± 1.3^a^	7.0 ± 1.2^b^	6.7 ± 1.7
Glucose AUC (mmol/l × min)	7.4 ± 1.5^a^	7.9 ± 0.8^b^	7.8 ± 1.3
Fasting insulin (pmol/l)	145.2 ± 53.5^a^	118.1 ± 43.1^c^	113.2 ± 35.4
Insulin AUC (pmol/l × min)	732.1 ± 373.8^a^	688.6 ± 493.1^c^	574.3 ± 202.7
HOMA-IR (AU)	4.9 ± 2.0^a^	4.1 ± 1.6^c^	3.9 ± 1.3
NEFA (mmol/l)	0.32 ± 0.13^a^	0.42 ± 0.16^c^	0.34 ± 0.18
NEFA AUC (mmol/l × min)	0.15 ± 0.06^a^	0.17 ± 0.06^c^	0.16 ± 0.07
ISI (AU)	42.5 ± 20.7^a^	43.4 ± 15.4^d^	48.3 ± 30.8
Insulinogenic index (AU)	35.7 ± 27.1^a^	20.2 ± 10.7^d^	21.4 ± 11.2^e^
DI (AU)	1453 ± 1422^a^	861 ± 575^d^	919 ± 420^e^

Values for continuous variables are presented as mean±SD, unless stated otherwise

^a^*n*=18

^b^*n*=15

^c^*n*=12

^d^*n*=10

^e^*n*=16

AU, arbitrary units; AUC, AUC from OGTT; DI, disposition index

### Responses to the OGTT (primary outcomes)

**Fig. 2:**
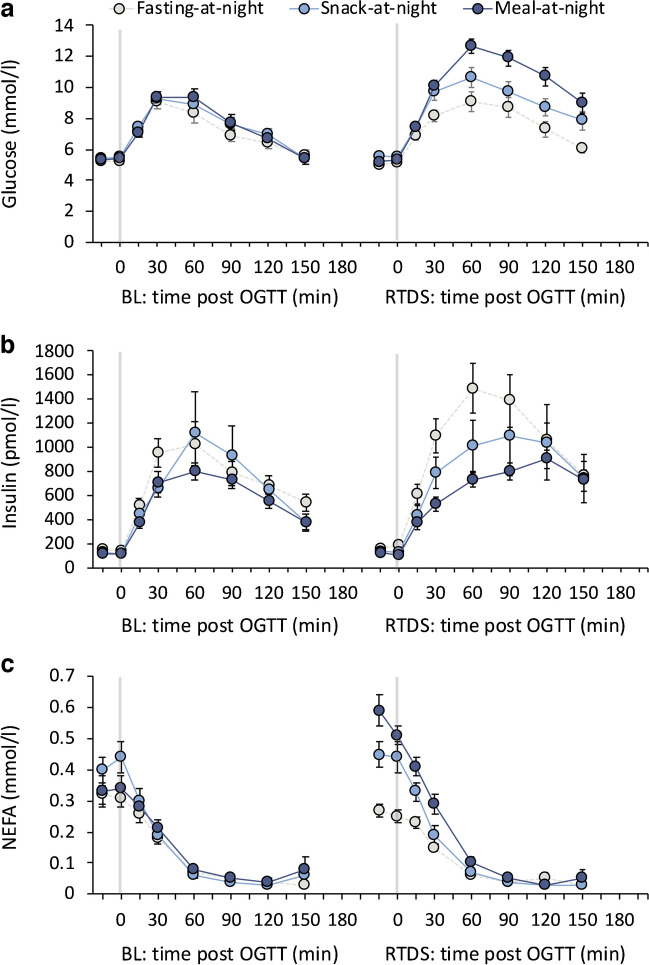
(**a**–**c**) Postprandial plasma glucose, insulin and NEFA in response to a 75 g OGTT at baseline (BL) and after 4 nights of simulated night-shift work plus 1 night of sleep to RTDS at Time = 0, 30, 60, 90, 120 and 150 min. Presented as means and SEM

For glucose AUC, there were condition×day (*p*<0.001), condition (*p*=0.018) and day (*p*<0.001) effects. Compared with baseline, glucose AUC was higher at RTDS in the meal-at-night (*p*<0.001) and snack-at-night (*p*<0.01) conditions, but not the fasting-at-night (*p*=0.220) condition (Table 2, Fig. 2). Post hoc comparison at RTDS revealed that glucose AUC was lower in the fasting-at-night condition vs snack-at-night (*p*=0.022) and meal-at-night (*p*<0.001) conditions. The results were similar for 2 h glucose values (Table 2). For insulin AUC, a condition-only effect was observed (*p*=0.047), with higher insulin AUC in the fasting-at-night condition vs other conditions (data not shown). For NEFA, there was a condition×day (*p*=0.001) and a condition (*p*=0.024) effect, with higher AUC from baseline in the meal-at-night (*p*<0.001), but not the snack-at-night (*p*=0.700) or fasting-at-night condition (*p*=0.146) (Table 2). Post hoc comparison at RTDS revealed NEFA AUC was lower in the fasting-at-night vs snack-at-night (*p*=0.045) and meal-at-night (*p*<0.001) conditions. For ISI, there was a main effect of day only (*p*=0.016; Table 2), with lower ISI on RTDS compared with baseline. For the insulinogenic index, a condition×day interaction (*p*=0.037) and a condition effect (*p*<0.001) was observed, with higher values from baseline in the fasting-at-night condition (*p*=0.030). Post hoc comparison at RTDS revealed that ISI for the meal-at-night condition was lower than snack-at-night (*p*=0.046) and fasting-at-night (*p*<0.001) conditions. For the disposition index, there was a main effect of condition (*p*=0.011; Table 2), being higher in the fasting-at-night vs meal-at-night condition (*p*=0.004) but with the difference not reaching statistical significance for the snack-at-night condition (*p*=0.052, data not shown). While not significant, there was also a reduction in disposition index from baseline in the meal-at-night condition only (*p*=0.052).

**Table 2:** Change in fasting and postprandial glucose, insulin and NEFA indices in response to an OGTT conducted at baseline and following RTDS after 4 nights of simulated night-shift work

Variable	*p* value (*F*_*df*_)			Estimated change from BL (95% CI)		
	Condition	Day	Condition× day	Fasting-at-night	Snack-at-night	Meal-at-night
Glucose AUC (mmol/l × min)	0.018 (*F*_2,47_=4.4)	<0.001 (*F*_1,47_=46.0)	<0.001^†^ (*F*_2,47_=9.5)	0.34 (−0.21, 0.89)	0.96 (0.36, 1.56)**	2.00 (1.45, 2.56)***
2 h glucose (mmol/l)	0.006 (*F*_2,47_=5.8)	<0.001 (*F*_1,47_=63.6)	<0.001^‡^ (*F*_2,47_=11.0)	0.89 (−0.02, 1.80)	1.77 (0.76, 2.76)**	3.87 (2.93, 4.80)***
Insulin AUC (pmol/l × min)	0.047 (*F*_2,46_=3.3)	0.111 (*F*_2,46_=2.6)	0.316 (*F*_2,46_=1.2)	262.5 (22.5, 502.5)*	112.1 (−195.6, 419.8)	−0.5 (−250.0, 40.8)
NEFA AUC (mmol/l × min)	0.024 (*F*_2,44_=4.1)	0.099 (*F*_1,44_=2.9)	0.001^†^ (*F*_2,44_=8.0)	−0.02 (−0.06, 0.01)	0.01 (−0.03, 0.05)	0.07 (0.03, 0.10)***
ISI (AU)	0.576 (*F*_2,42_=0.6)	0.016 (*F*_1,42_=6.3)	0.932 (*F*_2,42_=0.1)	−8.85 (−19.94, 2.24)	−7.39 (−22.27, 7.49)	−10.76 (−22.17, 0.65)
Insulinogenic index (AU)	0.001 (*F*_2,41_=5.7)	0.147 (*F*_1,41_=0.02)	0.037^§^ (*F*_2,41_=3.9)	13.3 (1.6, 25.1)*	11.7 (−4.1, 27.4)	−7.9 (−20.4, 4.5)
DI (AU)	0.011 (*F*_2,41_=5.0)	0.400 (*F*_1,41_=0.72)	0.230 (*F*_2,41_=1.5)	31.4 (−388.7, 451.5)	59.1 (−504.5, 622.7)	−441.0 (−886.6, 4.6)
Fasting glucose (mmol/l)	0.111 (*F*_2,47_=2.3)	0.987 (*F*_1,47_<0.1)	0.373 (*F*_2,47_=1.0)	−0.16 (−0.44, 0.12)	0.12 (−0.19, 0.42)	0.04 (−0.25, 0.33)
Fasting insulin (pmol/l)	0.043 (*F*_2,46_=3.4)	0.199 (*F*_1,45_=1.7)	0.429 (*F*_2,45_=0.9)	27.0 (−3.2, 57.2)	12.2 (−27.0, 51.3)	−0.1 (−32.6, 30.0)
HOMA-IR (AU)	0.077 (*F*_2,44_=2.7)	0.250 (*F*_1,43_=1.4)	0.657 (*F*_2,43_=0.4)	−0.63 (−1.62, 0.36)	−0.47 (−1.77, 0.82)	−0.00 (−1.02, 1.01)
Fasting NEFA (mmol/l)	<0.001 (*F*_2,44_=9.5)	0.024 (*F*_1,44_=5.4)	<0.001^†^ (*F*_2,44_=10.1)	−0.05 (−0.14, 0.03)	0.03 (−0.08, 0.13)	0.21 (0.13, 0.30)***

*p* values (with *F* values and *df*) are derived from mixed-effects ANOVA. For significant condition×day interactions, significant within-participant changes from baseline are indicated; *p<0.05, **p<0.01, ***p<0.001

^†^*p*<0.05 for between-condition post hoc comparisons at RTDS: fasting-at-night<snack-at-night and meal-at-night

^‡^*p*<0.05 for between-condition post hoc comparisons at RTDS: fasting-at-night<snack-at-night<meal-at-night

^§^*p*<0.05 for between-condition post hoc comparisons at RTDS: meal-at-night<snack-at-night and fasting-at-night

AU, arbitrary units; AUC, AUC from OGTT; BL, baseline; DI, disposition index

### Overnight fasting plasma glucose, insulin and NEFA

There were no main or interaction effects for fasting glucose (Table 2). For fasting insulin, there was an effect of condition only (*p*=0.043; Table 2), such that insulin was higher in the fasting-at-night condition relative to the meal-at-night condition (*p*=0.016, data not shown). For fasting NEFA, there were condition×day (*p*<0.001) and condition (*p*<0.001) effects, with higher values on RTDS vs baseline in the meal-at-night condition (*p*<0.001), but not the snack-at-night (*p*=0.627) or fasting-at-night (*p*=0.215) conditions. Post hoc comparison at RTDS revealed that NEFA levels were lower in the fasting-at-night condition vs snack-at-night (*p*=0.001) and meal-at-night (*p*<0.001) conditions (Table 2).

### Responses to a breakfast meal

**Fig. 3:**
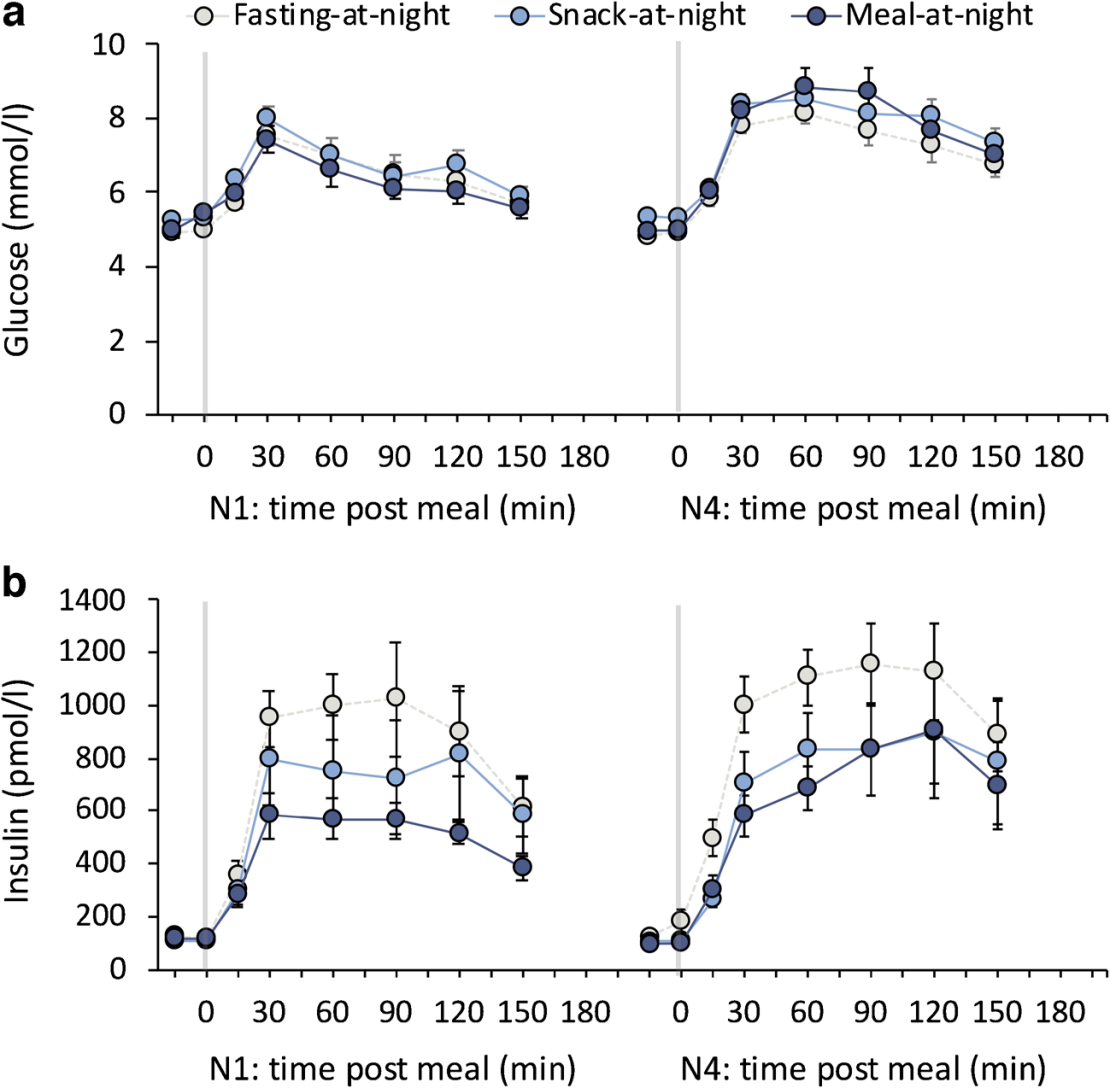
(**a**, **b**) Postprandial glucose and insulin in response to identical high carbohydrate (76%) breakfast meals given in the morning immediately following NS1(N1) and NS4 (N4) at Time = 0, 30, 60, 90, 120 and 150 min. Presented as means and SEM

Postprandial glucose and insulin AUCs, and calculations of insulin secretion and sensitivity in response to a breakfast meal tolerance test after NS1 by condition are given in ESM Table 1. For glucose AUC, there was a condition×day interaction (*p*=0.017) and day effect (*p*<0.001) as shown in Table 3 and Fig. 3. Glucose AUC was higher the morning after NS4 compared with NS1 in each condition (*p*<0.002; Table 3), but post hoc comparisons did not detect a difference between conditions. For insulin AUC, a significant effect of day was observed (*p*=0.025; Table 3), being higher after NS4 as compared with NS1. For ISI, there was a main effect of day only (*p*<0.001), such that values were lower after NS4 compared with NS1. For the insulinogenic index, there was a significant effect of condition only (*p*=0.032; Table 3), such that values were higher in fasting-at-night vs meal-at-night condition (*p*=0.009, data not shown). For disposition index, there was a significant effect of day only (*p*=0.008; Table 3), such that disposition index was lower on NS4 compared with NS1 (data not shown).

**Table 3:** Change in postprandial glucose and insulin in response to identical high carbohydrate breakfast meals provided in the morning after 1 night (NS1) and 4 nights (NS4) of simulated night-shift work

Variable	*p* value (*F*_*df*_)			Estimated change from NS1 (95% CI)		
	Condition	Day	Condition×day	Fasting-at-night	Snack-at-night	Meal-at-night
Glucose AUC (mmol/l × min)	0.528 (*F*_2,46_=0.7)	<0.001 (*F*_1,46_=69.8)	0.017^a^ (*F*_2,46_=4.5)	0.63 (0.25, 1.02)**	0.81 (0.41, 1.22)***	1.45 (1.03, 1.87)***
Insulin AUC (pmol/l × min)	0.088 (*F*_2,42_=2.6)	0.025 (*F*_1,42_=5.4)	0.495 (*F*_1,42_=0.7)	109.0 (−2.8, 220.2)	20.1 (−111.1, 151.4)	116.0 (−10.4, 242.4)
ISI (AU)	0.322 (*F*_2,42_=1.2)	<0.001 (*F*_1,42_=25.5)	0.813 (*F*_1,42_=0.8)	−9.9 (−15.3, −4.6)	−8.5 (−14.9, −2.2)	−7.4 (−13.5, −1.3)
IGI (AU)	0.032 (*F*_2,42_=3.8)	0.097 (*F*_1,42_=2.9)	0.522 (*F*_2,42_=0.7)	−4.3 (−20.9, 12.3)	−17.7 (−37.3, 1.8)	−4.9 (−24.0, 14.3)
DI (AU)	0.279 (*F*_2,43_=1.3)	0.008 (*F*_1,43_=7.9)	0.678 (*F*_2,43_=0.4)	−671 (−1584, 242)	−1191 (−2266, 117)	−588 (−1643, 467)

*p* values (with *F* values and *df*) are derived from mixed-effects ANOVA

^a^Since condition×day interaction is significant, between-condition post hoc comparisons at RTDS were conducted. There were no significant differences between conditions at NS4 vs NS1 following post hoc analysis

AU, arbitrary units; AUC, AUC from breakfast meal; DI, disposition index

### Sleep assessment

For all variables except for stage 1 sleep, a main effect of day only was observed (Table 4). Post hoc comparisons showed less TST and stage 3 sleep on NS3 and RTDS relative to baseline, but faster sleep onset on NS3 vs BL vs RTDS (Table 4). Stage 2 sleep was lower at NS3 vs RTDS and baseline. Participants displayed more WASO, reduced sleep efficiency, and less stage 4 and REM sleep at RTDS vs baseline and NS3 (Table 4).

**Table 4:** PSG measures of sleep at baseline, following NS3 and RTDS

Variable/time point	Condition (mean±SD)			*p* value (*F,*_*df*_)		
	Fasting-at-night	Snack-at-night	Meal-at-night	Condition	Day	Condition×day
TST (h)				0.926 (*F*_2,98_=0.1)	<0.001 (*F*_1,98_=25.9)^†^	0.654 (*F*_2,98_=0.6)
BL	7.2 ± 0.4	7.0 ± 0.5	7.1 ± 0.7			
NS3	5.8 ± 1.1	6.3 ± 0.6	6.0 ± 1.1			
RTDS	5.8 ± 1.9	5.7 ± 1.7	5.8 ± 1.2			
SOL (min)				0.474 (*F*_2,97_=0.8)	<0.001 (*F*_1,97_=25.7)^‡^	0.852 (*F*_2,97_=0.3)
BL	17.7 ± 12.3	29.9 ±23.1	19.3 ± 14.9			
NS3	8.4 ± 14.7	6.0 ± 3.9	4.8 ± 3.1			
RTDS	52.3 ± 72.6	60.7 ± 55.3	44.9 ± 37.9			
WASO (min)				0.861 (*F*_2,98_=0.2)	<0.001 (*F*_1,98_=9.3)^§^	0.269 (*F*_2,98_=1.3)
BL	29.8 ± 18.6	26.9 ±15.9	32.7 ±32.5			
NS3	65.1 ± 57.8	34.7 ± 34.9	41.8 ± 54.2			
RTDS	59.4 ± 64.4	78.3 ± 95.2	86.4 ± 86.6			
SE (%)				0.814 (*F*_2,98_=0.2)	<0.001 (*F*_1,98_=28.1)^¶^	0.501 (*F*_2,98_=0.8)
BL	90.1 ± 4.7	88.1 ± 6.2	89.1 ± 8.4			
NS3	82.5 ± 15.7	90.3 ± 8.8	88.8 ± 12.9			
RTDS	72.1 ± 23.2	70.9 ± 21.2	72.7 ± 14.7			
Stage 1 (min)				0.519 (*F*_2,98_=0.7)	0.115 (*F*_1,98_=2.2)	0.770 (*F*_2,98_=0.5)
BL	5.5 ± 4.2	6.6 ± 5.2	8.1 ± 6.9			
NS3	5.2 ± 3.5	6.6 ± 3.8	6.7 ± 4.7			
RTDS	7.2 ± 5.9	7.8 ± 5.7	7.8 ± 4.8			
Stage 2 (min)				0.823 (*F*_2,98_=0.2)	<0.001 (*F*_1,98_=24.5)^Ψ^	0.656 (*F*_2,98_=0.7)
BL	159.9 ± 34.9	161.3 ± 57.3	164.7 ± 49.9			
NS3	105.7 ± 36.7	122.5 ± 43.6	112.4 ± 39.6			
RTDS	148.3 ± 62.5	144.9 ± 67.1	161.1 ± 46.9			
Stage 3 (min)				0.827 (*F*_2,98_=0.1)	<0.001 (*F*_1,98_=19.7)^†^	0.513 (*F*_2,98_=0.8)
BL	85.0 ± 33.2	79.4 ± 32.5	77.3 ± 24.6			
NS3	54.8 ± 21.7	64.3 ± 21.5	59.9 ± 21.9			
RTDS	60.2 ± 35.7	62.8 ± 45.1	56.4 ± 23.4			
Stage 4 (min)				0.760 (*F*_2,98_=0.3)	<0.001 (*F*_1,98_=98.7)^¶^	0.386 (*F*_2,98_=0.4)
BL	86.9 ± 28.2	85.0 ± 46.2	79.9 ± 34.0			
NS3	88.2 ± 29.4	98.8 ± 41.9	83.7 ± 41.4			
RTDS	47.8 ± 28.8	42.1 ± 26.8	40.8 ± 24.3			
REM (min)				0.709 (*F*_2,98_=0.3)	0.049 (*F*_1,98_=3.1)^¶^	0.933 (*F*_2,98_=0.2)
BL	93.6 ± 19.9	90.4 ± 20.2	96.7 ± 31.4			
NS3	92.8 ± 29.6	86.4 ± 20.2	97.2 ± 33.8			
RTDS	82.6 ± 34.3	82.7 ± 30.6	82.7 ± 24.9			

*p* values (with *F* values and *df*) are derived from mixed-effects ANOVA

^†^*p*<0.05 for between-time-point post hoc comparisons: BL>NS3, RTDS

^‡^*p*<0.05 for between-time-point post hoc comparisons: NS3<baseline<RTDS

^§^*p*<0.05 for between-time-point post hoc comparisons: RTDS>baseline and NS3

^¶^*p*<0.05 for between-time-point post hoc comparisons: RTDS<baseline and NS3

^Ψ^* p*<0.05 for between-time-point post hoc comparisons: NS3<baseline and RTDS

BL, baseline; SE, sleep efficiency; SOL, sleep-onset latency

## Discussion

The potential for modified meal timing as a therapeutic strategy to prevent chronic disease has rarely been examined under controlled conditions during simulated night-shift work in adults. In this study, we found that individuals who ate either a meal or a snack at 00:30 hours during simulated nightwork displayed reduced glucose tolerance. Those who fasted at night displayed compensatory hyperinsulinaemia and maintained glucose tolerance. The study indicates that meal timing is an effective intervention to mitigate impairments in glucose metabolism due to night-shift work. To understand the effect of meal size as well as timing we also examined the effects of a snack on glucose metabolism. While a snack may be more tolerable for the individual than fasting overnight, glucose tolerance was still impaired in the snack-at-night vs fasting-at-night condition. It appears therefore, that avoiding eating at night is an important strategy for mitigating the impacts of night-shift work on glucose metabolism.

Multiple studies have previously shown that disruptions in circadian rhythms, such as those caused by night-shift work or exposure to light at night, impair insulin sensitivity [32–35]. Two of these studies measured peripheral insulin sensitivity by the clamp technique [32, 35] and another study showed that insulin sensitivity in response to breakfast and dinner meals was impaired in chronic shift workers after three night shifts vs three day shifts [33]. In all studies, individuals were provided with meals during the night shift. As a result, there was co-exposure to wakefulness, light at night and nutrients. The current study shows that removing nutrients at night did not rescue insulin sensitivity, although gold-standard methods to assess insulin resistance were not performed. Of note, in the context of this study and previous trials [32, 33], night-shift work did not appear to alter HOMA-IR, but caution should be exercised in using HOMA-IR as an accurate measure of total-body, peripheral or hepatic insulin resistance [36].

The mechanisms underpinning an acute induction in insulin resistance following night-shift work are likely to be multifactorial. Sleep restriction and higher levels of melatonin in the morning are both linked with poorer insulin sensitivity [37, 38]. In the current study, all participants were provided with a 7 h sleep opportunity following each night shift and, thus, sleep was not restricted. However, there were significant reductions in duration and quality of sleep across all conditions, which were clearly indicative of the effects of shiftwork on both circadian and sleep systems. Elevations in plasma NEFA also induce insulin resistance [39]; however, plasma NEFA levels were elevated solely in the meal-at-night condition, suggesting that these are unlikely to be a contributing factor in the development of insulin resistance across all three conditions. Disturbances in cortisol rhythms also occur in response to night-shift work [40] and are linked with impaired insulin sensitivity [41] and, therefore, may also play a role in this study.

When insulin resistance is present, normally functioning pancreatic beta cells increase the secretion of insulin to maintain blood glucose levels. In the current study, glucose tolerance was maintained solely in the fasting-at-night condition, potentially through an increase in insulin secretion, as measured by the insulinogenic index. In contrast, glucose tolerance deteriorated by 11–25%, respectively, in the snack-at-night and meal-at-night conditions, with the meal-at-night condition increasing 2 h glucose levels by 3.9 mmol/l. Although not akin to shiftwork, the findings of this study are consistent with a previous trial that showed that holding a daytime eating pattern during four 28 h ‘days’ prevented impairments in first-phase insulin secretion and glucose tolerance [16]. Together these studies show that fasting at night is more beneficial for insulin secretion and glucose control, although longer studies are needed to determine the longevity of effect.

Disposition index reflects the hyperbolic relationship between insulin secretion and sensitivity, with a reduced disposition index indicating a closer path towards diabetes [31]. In the current study, disposition index was not differentially altered by condition, although a trend (*p*=0.052) towards reduced disposition index in response to simulated night-shift work was observed in the meal-at-night condition. A previous study has shown that night-shift work impaired insulin secretion and disposition index as assessed by the C-peptide minimal model [42]. In that study, nurses worked two consecutive day or night shifts before testing in the day or night, respectively, and, so it cannot be determined whether the differences observed were due to natural circadian variation or the shiftwork itself. Since C-peptide was not assessed in the current study, we acknowledge that changes in insulin clearance, not insulin secretion, could also explain the effects that we observed.

Insulin secretion and hepatic clearance of insulin are both known to be under circadian regulation, with insulin secretion and glucose tolerance being higher and insulin clearance being lower in the morning as compared with the evening [43, 44]. Studies have shown that the peripheral clocks located within pancreatic islets cyclically regulate insulin secretion [45]. Additional experiments in rodents have revealed that the circadian clock in pancreatic beta cells directly controls the expression of key genes involved in insulin secretion, such as genes encoding glucose transporter 2 [46]. Moreover, disruption of circadian rhythms by chronic light exposure impairs beta cell function in mice and results in the loss of rhythmic islet gene expression and diurnal chromatin accessibility [47]. Importantly, this study also showed beta cell function was restored by TRE [47]. Impaired insulin secretion has also been associated with disruptions in melatonin rhythm, triggered by night-time light exposure [48], and melatonin administration in the morning reduced insulin secretion [37]. We speculate, therefore, that fasting during a night shift enables the maintenance of pancreatic peripheral clocks, whereas food intake impairs beta cell recovery and has an impact on the insulin secretory response [49]. Elevations in morning plasma NEFA were also observed in the snack-at-night and meal-at-night conditions vs the fasting-at-night condition upon RTDS in the current study. While elevated NEFA levels are known to stimulate insulin secretion in vitro, this is dependent on the type of fatty acid used in experiments [50] and high systemic NEFA levels are also linked with impaired insulin secretion [51].

While fasting at night prevented impairments in glucose metabolism, the acceptability of a complete overnight fast in night-shift workers is not well established [18]. Therefore, we also tested the effects of reducing the size of the meal during the night shift. While consumption of a snack appeared to have an intermediary effect, glucose tolerance was still impaired in the snack-at-night vs fasting-at-night conditions. It should be noted that only the energy content of the snack was manipulated and we did not alter macronutrient content. Replacing carbohydrates with protein could have resulted in a different outcome.

The strengths of this study are that it simulated real-world, night-shift work conditions in a controlled laboratory environment in healthy individuals who had not previously been exposed to regular night-shift work. Importantly, the GTT was performed after a return to day-shift schedule, meaning that all participants had identical fasting lengths prior to testing. While we additionally performed a breakfast meal challenge after the first and fourth night shift, there were marked differences in the fasting lengths before breakfast due to the protocol design, which limits the interpretation of the data. We did not assess C-peptide, which is secreted in equimolar amounts with insulin and avoids hepatic degradation, thus providing a better indication of insulin secretory capacity. The study was not powered to analyse by sex and so this analysis was not conducted. The findings from this study are limited to healthy, non-shift working adults without obesity or metabolic disease.

The study was performed under simulated conditions, but fasting at night may also be effective for shift workers under real-world conditions [52], although one study showed that fasting overnight also increased food intake the following day [53]. Future studies could consider manipulating the carbohydrate content of late-night snacks to determine whether eating a protein-enriched snack would also prevent deteriorations in glucose tolerance. Optimal meal timing may also be a moving target across the number of nights that shift work is performed; thus, further studies could also determine whether night-shift workers should adjust meal timing, daily, in accordance with a shift in central and peripheral clocks.

To date, the potential of manipulating meal timing as a therapeutic strategy to prevent chronic disease has rarely been examined in night-shift working adults. The current study clearly shows that simulated night-shift work induced insulin resistance, which was not rescued by altering either meal timing or meal size. However, insulin secretion was increased in the fasting-at-night condition, which prevented acute deteriorations in glucose tolerance. Meal timing advice should be considered for existing dietary guidelines, industry recommendations and workplace policy to improve health and reduce the burden of metabolic disease on night-shift workers.

## Supplementary Information

Below is the link to the electronic supplementary material.

ESM (PDF 285 KB)

## Data Availability

Data are not publicly available as they contain information that could compromise research participant consent. Anonymised data from this study will be made available upon reasonable request from the corresponding author, with a full research plan for academic use, only for a period of 36 months from the date of publication.

## Abbreviations

ANZCTR: Australian New Zealand Clinical Trial Registry
CONSORT: Consolidated Standards of Reporting Trials
EER: Estimated energy requirements
IGI: Insulinogenic beta cell index
ISI: Insulin sensitivity index
NS1–4: Night shift 1–4
PSG: Polysomnography
REM: Rapid eye movement
RTDS: Return to a daytime schedule
TIB: Time-in-bed
TRE: Time-restricted eating
TST: Total sleep time
WASO: Wake after sleep onset
