# Supplementary material for: Fasting as an intervention to alter the impact of simulated night-shift work on glucose metabolism in healthy adults: a cluster randomised controlled trial
Source: Diabetologia. 2024 Oct 18;68(1):203–16. doi: 10.1007/s00125-024-06279-1 (PMC11663163; doi:10.1007/s00125-024-06279-1)
Supplement: Supplementary file 1 — ESM (PDF 285 KB) [file 125_2024_6279_MOESM1_ESM.pdf]

Electronic Supplementary Material (ESM)

ESM Table 1. Postprandial glucose and insulin area under curves in response to a breakfast meal tolerance test and calculated insulin sensitivity, insulinogenic index, and disposition index by condition after nightshift 1.

	Fasting-at-night	Snack-at-night	Meal-at-night
Glucose AUC (mmol/L/min)	6.4± 1.1	6.7±0.9	6.2±0.9
Insulin AUC (pmol/L/min)	754.2±421.8	598.3±553.9	424.6±161.9
Insulin Sensitivity Index (AU)	48.7±23.3	57.2±27.3	57.0±17.9
Insulinogenic Index (AU)	48.1±20.0	47.8±47.3	29.2±30.3
Disposition Index (AU)	1565.9±663.6	1334.7±674.8	1082.3±559.3

Note: Values for continuous variables represent averages ± standard deviation, AUC = area under curve in response to breakfast meal tolerance test.

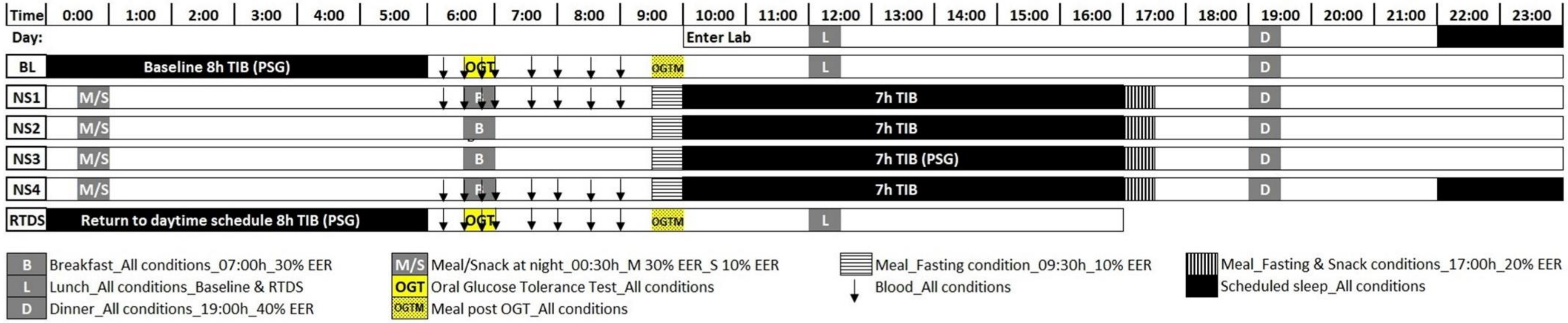

ESM Figure 1. Laboratory protocol depicting laboratory testing and the meal, shiftwork and sleep opportunities provided to participants by condition.

Note: Time of day is indicated along the top of the figure. Day is indicated on left hand side of figure (BL=Baseline, NS1–NS4 = Nightshifts 1-4, RTDS = Return to daytime schedule). Meals for all conditions convey timing and percent of daily energy intake for each meal. Black boxes show scheduled sleep opportunities (PSG denotes sleep periods where sleep was measured using polysomnography). Yellow boxes show 75 gram oral glucose tolerance test (OGTT) on BL and RTDS days (OGTT constitutes approx. 10% of daily energy). Black arrows show when blood was taken during OGTT and breakfast meal tolerance (MT) timepoints on NS1 and NS4.
